# Sex hormone balance and PSA-derived biomarkers in cancer-free African migrant men in Italy: Implications for prostate cancer early detection

**DOI:** 10.1016/j.jlb.2026.100478

**Published:** 2026-06-26

**Authors:** Felice Crocetto, Matteo Ferro, Gianluigi Carbone, Mariano Fiorenza, Rosa Sirica, Maria Perito, Carmela Polito, Michele Cennamo, Francesco Del Giudice, Daniela Terracciano

**Affiliations:** a Department of Neuroscience, Reproductive and Odontostomatological Sciences, University of Naples “Federico II”, Naples, Italy; b Unit of Urology, Department of Health Science, ASST Santi Paolo and Carlo, University of Milan, Milan, Italy; c Department of Translational Medical Sciences, University of Naples “Federico II”, Naples, Italy; d Giudice Department of Maternal, Infant and Urological Sciences, Sapienza University of Rome, Policlinico Umberto I Hospital, Rome, Italy

**Keywords:** Prostate cancer, PHI, PSA, Ethnicity, Black men, Estradiol, Testosterone

## Abstract

**Background:**

Men of African ancestry experience a disproportionate burden of prostate cancer (PCa), with higher incidence and mortality than men of European ancestry. Although social determinants of health, barriers to care, and delayed diagnosis are key contributors to these disparities, ancestry- and context-related differences in sex hormone milieu and PCa-associated laboratory biomarkers remain insufficiently characterized. We investigated sex hormone profiles, PSA molecular forms, and derived hormone-to-biomarker ratios in cancer-free African migrant men living in Italy compared with White controls.

**Methods:**

This single-center prospective observational study included 87 cancer-free men: 40 Black men of African origin and 47 White controls. Serum total prostate-specific antigen (PSA), testosterone, estradiol, and the testosterone-to-estradiol ratio were compared between groups. Free PSA, [-2]proPSA, and the Prostate Health Index (PHI) were assessed in the subset of participants with available PSA molecular form measurements. Exploratory hormone-to-biomarker ratios were calculated by relating testosterone, estradiol, and the testosterone-to-estradiol ratio to total PSA, free PSA, [-2]proPSA, and PHI.

**Results:**

Total PSA levels did not significantly differ between Black men of African origin and White controls (median 1.22 vs. 1.10 ng/mL; P = 0.407). Testosterone concentrations were also comparable between groups (median 468.48 vs. 412.88 ng/dL; P = 0.362). Conversely, estradiol concentrations were significantly higher in Black men of African origin (median 32.29 vs. 21.53 pg/mL; P < 0.001; q < 0.001), while the testosterone-to-estradiol ratio was significantly lower (median 12.85 vs. 20.20; P < 0.001; q < 0.001). PHI and [-2]proPSA were significantly lower in Black men of African origin than in White controls (PHI: median 27.06 vs. 52.72, P = 0.004, q = 0.012; [-2]proPSA: median 8.90 vs. 23.80 pg/mL, P = 0.008, q = 0.021). Estradiol-to-PSA molecular-form ratios were consistently higher in Black men of African origin, particularly estradiol/free PSA, estradiol/[-2]proPSA, and estradiol/PHI, whereas the (testosterone-to-estradiol ratio)/total PSA was lower.

**Conclusions:**

Cancer-free African migrant men living in Italy showed a distinct biochemical phenotype characterized by higher estradiol levels and a lower testosterone-to-estradiol ratio, despite comparable total PSA and testosterone levels. The concomitantly lower PHI and [-2]proPSA values, together with higher estradiol-to-PSA-derived biomarker ratios, suggest that sex hormone balance may influence the context-aware interpretation of PCa biomarkers in this population.

## 1 Introduction

Prostate cancer (PCa) remains one of the most frequent malignancies among men worldwide and represents a major cause of cancer-related morbidity and mortality [1]. Its incidence has increased over recent decades, partly because of population ageing, improved diagnostic pathways and widespread use of prostate-specific antigen (PSA)-based testing [2]. Although age and family history are well-established risk factors, ethnicity has emerged as a major determinant of PCa risk, clinical presentation and outcomes [3].

Men of African ancestry are disproportionately affected by PCa [4,5]. In several Western countries, including the United States, the United Kingdom and parts of Europe, Black men have been reported to experience higher PCa incidence and mortality compared with White men [6,7]. They are also more likely to be diagnosed at a younger age and, in some settings, with more aggressive disease. The reasons underlying these disparities are complex and multifactorial, involving genetic susceptibility, hormonal milieu, environmental exposures, lifestyle factors, socioeconomic inequalities, reduced access to healthcare, delayed diagnosis, lower health literacy and structural barriers within healthcare systems [6,8,9].

Although PSA-based testing remains a cornerstone of PCa early detection, its interpretation is constrained by limited cancer specificity and variable performance across clinical contexts. Total PSA may lead to unnecessary biopsies, overdiagnosis and overtreatment [10]. To improve diagnostic accuracy, several PSA-derived biomarkers and indices have been introduced, including the Prostate Health Index (PHI) [11]. PHI combines total PSA, free PSA and [−2]proPSA and has shown improved performance compared with total PSA alone for identifying clinically significant PCa [11]. However, most validation studies have been conducted in populations of predominantly European ancestry, and relatively little is known about whether PSA molecular forms and PHI behave similarly across ethnic groups [12].

Although emerging evidence has begun to address this gap, available data remains limited and not entirely consistent. In biopsy-naïve Black men, Babajide et al. [13] reported that PHI showed only moderate accuracy for detecting Gleason grade group 2–5 PCa, with performance comparable to PSA in the 4.0–10.0 ng/mL range; nevertheless, a PHI threshold of 28.0, when used in combination with PSA, reduced unnecessary biopsies while maintaining high sensitivity for clinically significant disease. More recently, Morris et al. [14] found that current PHI risk stratifications may have limited clinical utility in African American men meeting FDA-approved indications for PHI testing, with high rates of clinically significant PCa across conventional PHI categories and only a small proportion of biopsies avoided using a low PHI threshold. Together, these studies suggest that PHI interpretation may require population-specific validation and that conventional PHI thresholds may not be directly generalizable to Black men.

This knowledge gap is particularly relevant for Black men, for whom PCa screening and early detection represent major clinical and public health priorities [15]. On the one hand, timely diagnosis is essential to reduce the burden of advanced and lethal disease. On the other hand, inappropriate interpretation of laboratory biomarkers may lead to unnecessary diagnostic procedures or, conversely, to under-recognition of clinically relevant risk. Therefore, determining whether ancestry- and context-related differences exist in PSA-derived biomarkers is crucial for developing more accurate, equitable, and clinically appropriate diagnostic strategies.

In addition to PSA-related biomarkers, sex steroid hormones may contribute to PCa biology and disparities across ancestry groups. Testosterone and estradiol are involved in prostate development, homeostasis, and carcinogenesis, while an altered androgen-to-estrogen balance may influence PCa risk, progression, and tumor–stroma interactions. In this context, integrating PSA-derived biomarkers with measures of sex hormone balance may provide a more comprehensive view of PCa risk-related biochemical profiles [16]. Consistently, emerging evidence suggests that ancestry-associated differences in prostate stromal estrogen signaling may be biologically relevant in PCa. Moreover, estrogen receptor (ER) signaling and estrogen-related receptor alpha (ERRα)-dependent metabolic programs have been implicated in PCa biology, tumor progression, and therapeutic vulnerability [[17], [18], [19], [20], [21], [22], [23], [24]].

In the present study, we aimed to compare total PSA, PSA molecular forms, PHI, testosterone, estradiol, and the testosterone-to-estradiol ratio in a cohort of cancer-free Black migrant men of African origin living in Italy and White controls. We also explored derived hormone-to-biomarker ratios to investigate the relationship between sex hormone balance and PSA-derived biomarkers in this population.

## 2 Materials and methods

### 2.1 Study design and participants

We conducted a single-center, prospective, observational study at the University of Naples Federico II, Naples, Italy. The study cohort included 87 cancer-free men, comprising 40 Black migrant men of African origin and 47 White control subjects.

All participants underwent clinical evaluation according to routine clinical practice, including collection of medical history, digital rectal examination (DRE), and prostate assessment. At enrolment, none of the participants had clinical evidence of PCa.

Exclusion criteria included a previous diagnosis of PCa, prior prostatectomy, current or previous hormonal therapy, anti-androgen treatment, treatment with luteinizing hormone-releasing hormone analogues or antagonists, and the use of medications potentially affecting prostate-specific antigen levels, including 5-alpha reductase inhibitors.

Written informed consent was obtained from all participants before enrolment. The study was conducted in accordance with the principles of the Declaration of Helsinki and was approved by the Ethics Committee of the University of Naples Federico II (Project identification code 118/20).

### 2.2 Laboratory measurements

Blood samples for serum total testosterone and estradiol measurement were collected in the morning, between 7:00 and 10:00 a.m. Hormone concentrations were determined using an automated Atellica analyzer (Siemens Healthcare Diagnostics, Tarrytown, NY, USA). For PSA-related biomarker assessment, blood samples were drawn before DRE. Serum was separated by centrifugation and stored at −80 °C until analysis, following established pre-analytical procedures to preserve [−2]proPSA stability. Total PSA, free PSA, and [−2]proPSA concentrations were measured in a blinded manner using the Access 2 Immunoassay System analyzer (Beckman Coulter Inc, Brea, CA, USA). PHI was calculated using the standard formula: PHI = ([−2]proPSA/free PSA) × √total PSA.

### 2.3 Derived hormone-to-biomarker ratios

To explore the interplay between sex hormone status and prostate-related biomarkers, a set of derived hormone-to-biomarker ratios was calculated. Total testosterone, estradiol, and the testosterone-to-estradiol ratio were each divided by total PSA, free PSA, [−2]proPSA, and PHI.

Ratio analyses were restricted to cases with complete data for both the numerator and denominator of each ratio, and no imputation of missing values was performed.

### 2.4 PSA molecular form analysis

PSA molecular forms and PHI were available in 28 participants, comprising 14 Black men of African origin and 14 White controls. This subset was used to compare free PSA, [−2]proPSA, the free-to-total PSA ratio, PHI, and derived hormone-to-biomarker ratios based on PSA molecular forms. Because not all participants with available PSA molecular form measurements had total PSA values within the conventional diagnostic grey zone, these analyses were interpreted as exploratory biomarker-distribution analyses rather than as a formal diagnostic-validation assessment.

### 2.5 Statistical analysis

Descriptive statistics were used to summarize the study population. Continuous variables are presented as median and interquartile range, together with the number of available observations.

Between-group differences were assessed using the two-sided Mann–Whitney *U* test, owing to the limited sample size and the skewed distribution of several biomarkers and derived ratios. Exploratory adjustment for multiple comparisons was performed using the Benjamini–Hochberg false discovery rate (FDR) procedure. A two-sided *P* value < 0.05 was considered statistically significant, and FDR-adjusted *q* values were reported to aid interpretation of exploratory analyses. Statistical analyses were performed using R software, version 4.0.3.

## 3 Results

### 3.1 Study population

The study cohort comprised 87 cancer-free men, including 40 Black men of African origin and 47 White controls. Black men of African origin were significantly younger than White controls (median age, 52.60 vs. 64.53 years; *P* = 0.005). Descriptive characteristics and primary biomarker comparisons are summarized in Table I.

**Table 1:** Primary clinical and biomarker comparisons between Black men of African origin and White controls.

Variable	Black men of African origin, median (IQR); n	White controls, median (IQR); n	P value	FDR-adjusted *q* value
Age, years	52.60 (45.64-58.22); n = 40	64.53 (48.80-69.39); n = 47	**0.005**	**0.015**
Total PSA, ng/mL	1.22 (0.76-2.24); n = 39	1.10 (0.56-2.78); n = 47	0.407	0.479
Free PSA, ng/mL	0.51 (0.26-0.92); n = 14	0.88 (0.67-1.18); n = 14	0.043	0.072
[-2]proPSA, pg/mL	8.90 (5.42-17.77); n = 14	23.80 (15.92-26.02); n = 14	**0.008**	**0.021**
PHI	27.06 (21.54-34.97); n = 14	52.72 (39.33-79.54); n = 14	**0.004**	**0.012**
Estradiol, pg/mL	32.29 (24.45-40.58); n = 39	21.53 (16.07-24.38); n = 47	**<0.001**	**<0.001**
Testosterone, ng/dL	468.48 (312.85-598.56); n = 39	412.88 (286.09-536.04); n = 47	0.362	0.453
Testosterone/Estradiol	12.85 (10.04-16.77); n = 39	20.20 (17.10-22.77); n = 47	**<0.001**	**<0.001**

Data are reported as median (interquartile range), together with the number of available observations. Between-group comparisons were performed using the two-sided Mann–Whitney *U* test. FDR-adjusted q values were calculated using the Benjamini–Hochberg procedure. PSA, prostate-specific antigen; PHI, Prostate Health Index; IQR, interquartile range; FDR, false discovery rate.

### 3.2 PSA and testosterone levels in the overall cohort

In the overall cohort, total PSA concentrations did not differ significantly between Black men of African origin and White controls (median, 1.22 vs. 1.10 ng/mL; *P* = 0.407). Circulating testosterone levels were also comparable between groups (median, 468.48 vs. 412.88 ng/dL; *P* = 0.362).

### 3.3 Estradiol levels and testosterone-to-estradiol balance

**Fig. 1:**
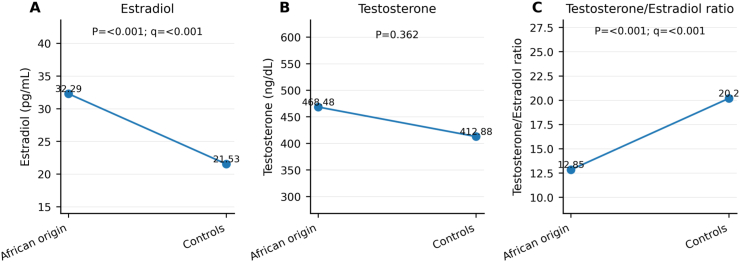
Distribution of circulating sex hormones and testosterone/estradiol balance in Black men of African origin and White controls.

Estradiol concentrations were significantly higher in Black men of African origin than in White controls (median, 32.29 vs. 21.53 pg/mL; *P* < 0.001; *q* < 0.001). Accordingly, the testosterone-to-estradiol ratio was significantly lower in Black men of African origin (median, 12.85 vs. 20.20; *P* < 0.001; *q* < 0.001), indicating a shift in sex hormone balance toward relatively higher estradiol levels (Fig. 1).

### 3.4 PSA molecular forms and PHI

**Fig. 2:**
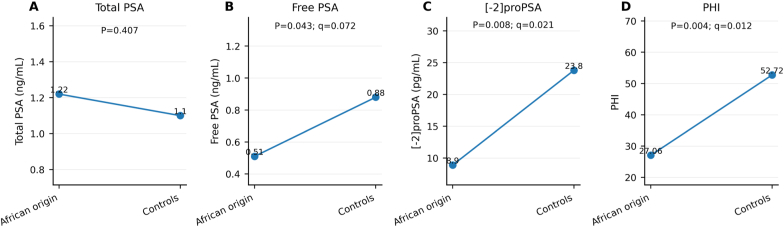
Distribution of total PSA, PSA molecular forms and PHI in Black men of African origin and White controls.

Among PSA-related biomarkers, free PSA was lower in Black men of African origin, although the difference showed only borderline significance after FDR correction (median, 0.51 vs. 0.88 ng/mL; *P* = 0.043; *q* = 0.072). [−2]proPSA concentrations were significantly lower in Black men of African origin (median, 8.90 vs. 23.80 pg/mL; *P* = 0.008; *q* = 0.021), as was PHI (median, 27.06 vs. 52.72; *P* = 0.004; *q* = 0.012) (Fig. 2).

### 3.5 Hormone-to-biomarker ratio analysis

**Fig. 3:**
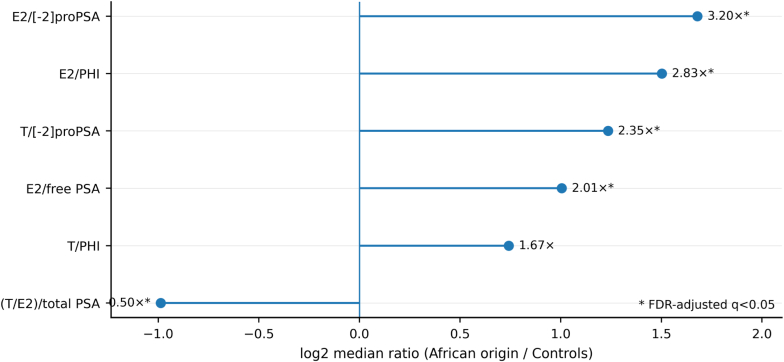
Direction and magnitude of hormone-to-biomarker ratio differences. Positive values indicate higher median ratios in Black men of African origin; negative values indicate lower median ratios. Asterisks indicate FDR-adjusted q < 0.05. T, testosterone; E2, estradiol; PSA, prostate-specific antigen; PHI, Prostate Health Index.

The hormone-to-biomarker ratio analysis is presented in Table II and Fig. 3. The most consistent between-group differences were observed for estradiol-based ratios. Estradiol/free PSA was significantly higher in Black men of African origin than in White controls (median, 50.38 vs. 25.10; *P* = 0.003; *q* = 0.010). Similarly, estradiol/[−2]proPSA was markedly higher in Black men of African origin (median, 3.17 vs. 0.99; *P* < 0.001; *q* = 0.003), as was estradiol/PHI (median, 1.19 vs. 0.42; *P* < 0.001; *q* < 0.001).

**Table 2:** Derived hormone-to-biomarker ratios according to study group.

Hormone-to-biomarker ratio	Black men of African origin, median (IQR); n	White controls, median (IQR); n	P value	FDR-adjusted *q* value
Testosterone/Total PSA	388.34 (169.28-594.19); n = 39	449.83 (165.06-669.46); n = 47	0.658	0.693
Estradiol/Total PSA	27.61 (10.76-59.92); n = 39	20.82 (6.78-36.99); n = 47	0.163	0.232
(Testosterone/Estradiol)/Total PSA	11.12 (5.95-17.13); n = 39	22.05 (8.20-35.50); n = 47	**0.016**	**0.036**
Testosterone/Free PSA	831.57 (555.56-2378.28); n = 14	474.70 (365.63-769.16); n = 14	0.113	0.174
Estradiol/Free PSA	50.38 (40.17-170.23); n = 14	25.10 (19.22-33.35); n = 14	**0.003**	**0.010**
(Testosterone/Estradiol)/Free PSA	32.70 (15.78-57.60); n = 14	20.12 (16.83-33.38); n = 14	0.696	0.696
Testosterone/[-2]proPSA	44.76 (24.98-76.94); n = 14	19.02 (16.56-26.73); n = 14	**0.020**	**0.041**
Estradiol/[-2]proPSA	3.17 (1.95-7.36); n = 14	0.99 (0.78-1.24); n = 14	**<0.001**	**0.003**
(Testosterone/Estradiol)/[-2]proPSA	1.47 (0.80-2.05); n = 14	0.92 (0.62-1.28); n = 14	0.323	0.431
Testosterone/PHI	13.18 (11.22-23.10); n = 14	7.88 (5.25-13.90); n = 14	0.037	0.066
Estradiol/PHI	1.19 (0.85-1.54); n = 14	0.42 (0.28-0.49); n = 14	**<0.001**	**<0.001**
(Testosterone/Estradiol)/PHI	0.50 (0.31-0.58); n = 14	0.42 (0.22-0.59); n = 14	0.448	0.498

Data are reported as median (interquartile range), together with the number of available observations. Between-group comparisons were performed using the two-sided Mann–Whitney *U* test. FDR-adjusted q values were calculated using the Benjamini–Hochberg procedure. PSA, prostate-specific antigen; PHI, Prostate Health Index; IQR, interquartile range; FDR, false discovery rate.

Testosterone/[−2]proPSA was also significantly higher in Black men of African origin than in White controls (median, 44.76 vs. 19.02; *P* = 0.020; *q* = 0.041). Conversely, the (testosterone/estradiol)/total PSA ratio was significantly lower in Black men of African origin (median, 11.12 vs. 22.05; *P* = 0.016; *q* = 0.036). Testosterone/PHI showed a nominally higher value in Black men of African origin (median, 13.18 vs. 7.88; *P* = 0.037), but this difference did not remain significant after FDR correction (*q* = 0.066).

## 4 Discussion

In this study, we found that cancer-free Black men of African origin exhibited a distinct circulating biochemical profile compared with White controls. In the overall cohort, estradiol concentrations were significantly higher and the testosterone-to-estradiol ratio was significantly lower in Black men of African origin, whereas total PSA and testosterone levels did not differ significantly between groups. In the subgroup with available PSA molecular form measurements, Black men of African origin also showed lower [−2]proPSA and PHI values. Furthermore, estradiol-based ratios relative to PSA molecular forms and PHI were consistently higher in Black men of African origin, suggesting that sex hormone balance may influence the baseline interpretation of PSA-derived biomarker profiles.

These findings extend our previous preliminary observations [25] in a smaller cohort of healthy Black men of African origin and White men, in which Black men showed lower PHI, lower [−2]proPSA/free PSA ratio, lower testosterone-to-estradiol ratio, and higher estradiol concentrations. The present study, based on a larger cohort and including an expanded hormone-to-biomarker ratio analysis, strengthens the hypothesis that ancestry- and context-related differences may exist not only in PCa incidence and outcomes, but also in the baseline distribution of laboratory biomarkers used for PCa risk assessment.

PCa disparities in men of African ancestry are well recognized and likely result from the complex interaction of biological, environmental, social, and healthcare-related determinants [26]. Black men have been reported to experience higher PCa incidence and mortality and, in some settings, to be diagnosed at a younger age and with more aggressive disease [3]. However, the mechanisms underlying these disparities remain incompletely understood. While genetic susceptibility, socioeconomic factors, access to care, health literacy, and delayed diagnosis have all been implicated [4], relatively little attention has been paid to whether commonly used laboratory biomarkers may behave differently across ancestry groups.

This point is clinically relevant. PSA-based testing remains central to PCa early detection, but total PSA has limited specificity [27]. PSA-derived biomarkers, including free PSA, [−2]proPSA, and PHI, have been developed to improve risk stratification and reduce unnecessary biopsies [28]. However, these tools have been mainly validated in populations of predominantly European ancestry, and available evidence in Black men suggests that their performance and optimal thresholds may differ across populations [13,14]. In biopsy-naïve Black men, Babajide et al. [13] reported that PHI had only moderate accuracy for detecting clinically significant PCa and was not superior to PSA in the diagnostic grey zone. Similarly, Morris et al. [14] showed limited clinical utility of conventional PHI risk stratifications in African American men, with high rates of clinically significant PCa across standard PHI categories and only a small proportion of biopsies avoided using a low PHI threshold. These findings support the need for population-specific validation of PSA-derived biomarkers and provide a clinical context for our observation that differences in PHI and PSA molecular forms may already be detectable at the cancer-free baseline level.

In our cohort, total PSA did not significantly differ between Black men of African origin and White controls. This finding suggests that the lower PHI and [−2]proPSA values observed in Black men of African origin were not simply driven by lower total PSA concentrations. Because PHI is calculated from total PSA, free PSA, and [−2]proPSA, the lower PHI values may reflect differences in the relative distribution of PSA molecular forms, particularly [−2]proPSA. One possible explanation is that ethnicity- or host context-related differences in PSA precursor processing within the prostate gland may influence PSA molecular profiles. However, this hypothesis remains speculative and requires mechanistic validation.

The finding of significantly higher estradiol levels and lower testosterone/estradiol ratio in Black men of African origin may be biologically meaningful. Sex steroid hormones are involved in prostate development, prostate function and prostate carcinogenesis [18]. Although testosterone has historically received most attention in PCa biology, increasing evidence supports a role for estrogens, ERs and estrogen-related receptor signaling in PCa initiation, progression, stromal biology, stemness and metabolic adaptation [18,[22], [23], [24]]. A lower androgen-to-estrogen balance may therefore represent one potential component of the biological background contributing to PCa susceptibility or aggressiveness in men of African ancestry. Overall, these findings support the concept that the interpretation of PCa biomarkers may be context-aware, with hormone-to-biomarker ratios potentially providing additional information on how sex hormone balance interacts with PSA-derived biomarker profiles.

Importantly, testosterone levels alone did not significantly differ between groups, whereas estradiol and the testosterone/estradiol ratio did. This suggests that the hormonal difference observed in Black men of African origin is not adequately captured by testosterone measurement alone. The testosterone/estradiol ratio may provide a more integrated representation of the circulating sex hormone balance and may deserve further investigation in PCa risk stratification studies.

The hormone-to-biomarker ratio analysis provides an additional exploratory perspective. Estradiol/free PSA, estradiol/[−2]proPSA and estradiol/PHI were markedly higher in Black men of African origin, whereas (testosterone/estradiol)/total PSA was lower. These results support the concept of a more estrogen-weighted biochemical profile relative to PSA-derived biomarkers. Whether this pattern reflects differences in endocrine milieu, prostate stromal biology, PSA molecular processing, body composition or other host-context factors cannot be established from the present dataset, but it provides a rationale for future studies integrating hormonal, inflammatory, metabolic and imaging-derived variables.

A theoretical PSA cut-off for accelerated early detection could not be reliably calculated from the present dataset because all participants were considered cancer-free and no biopsy-confirmed prostate cancer endpoint was available. Therefore, receiver operating characteristic curve analysis, Youden index estimation, or diagnostic threshold optimization would not be statistically meaningful in this cohort. Nevertheless, the observation that total PSA was comparable between groups despite differences in PHI, [−2]proPSA, and hormone-to-biomarker ratios suggests that future prospective studies should evaluate whether conventional PSA thresholds require recalibration in Black men of African origin. Indeed, as already emphasized in the literature [29], current PSA thresholds and secondary screening tools have not been adequately validated in Black men, and their clinical performance should be assessed not only in terms of discrimination, but also with respect to specificity, negative predictive value, and calibration. In this context, our findings further support the need for ancestry-aware and context-aware interpretation of PSA-derived biomarkers, aimed at improving early detection while minimizing unnecessary biopsies and overdiagnosis.

From a translational perspective, our findings suggest that PSA-derived biomarkers should not be interpreted as isolated laboratory values, but rather within a broader host-context framework. In clinical practice, this could support a risk-adapted pathway for Black men of African origin in which total PSA, PSA molecular forms, PHI, sex hormone balance, family history, digital rectal examination, prostate volume, and imaging findings are integrated rather than interpreted separately. Such an approach may help identify men who require closer follow-up or earlier referral while avoiding inappropriate reliance on uniform thresholds derived mainly from populations of European ancestry. However, the present study is exploratory and cancer-free by design; therefore, these observations require validation in larger prospective cohorts including biopsy and imaging outcomes before implementation in routine clinical decision-making.

In European countries, men of African origin may face multiple barriers to PCa prevention, including reduced awareness of PCa risk, linguistic and cultural barriers, limited access to preventive pathways and lower trust in healthcare systems [30]. In this context, the correct interpretation of laboratory tests should be integrated with culturally adapted prevention strategies and community-based approaches. Biomarker research and public health interventions should therefore be considered complementary dimensions of the same objective: reducing PCa inequalities [31,32].

Several limitations should be acknowledged. First, the sample size was relatively limited, particularly for analyses involving PSA molecular forms and PHI. Second, this was a single-centre study, and the findings may not be generalizable to all men of African ancestry, who represent a genetically, geographically, and culturally heterogeneous population. Third, participants were considered cancer-free based on available clinical assessment; however, the absence of PCa cannot be established with absolute certainty in the absence of systematic imaging and/or biopsy in all subjects. Fourth, age differed between groups and may have influenced some biomarker comparisons; therefore, larger age-matched cohorts are warranted. Fifth, the hormone-to-biomarker ratio analyses were exploratory and hypothesis-generating and should be validated in independent datasets before any clinical application.

The potential integration of molecular assessment also deserves consideration. Germline breast cancer susceptibility gene 1 and 2 (BRCA1/2) status was not available in the present cohort, and therefore we could not directly assess whether the observed hormone-to-biomarker patterns differed according to inherited DNA-repair gene alterations. Nevertheless, BRCA1/2 pathogenic variants are increasingly recognized as relevant determinants of prostate cancer risk, aggressiveness, and early detection strategies. Future studies in Black men of African origin should therefore evaluate PSA-derived biomarkers and sex hormone balance together with germline risk assessment, including BRCA1/2 and other DNA-repair genes, to determine whether ancestry, endocrine milieu, PSA molecular forms, and inherited molecular risk jointly improve identification of men who may benefit from earlier or more intensive diagnostic surveillance.

Despite these limitations, the study has several strengths. It addresses an underexplored area of laboratory medicine and PCa disparities and focuses on cancer-free men, allowing the assessment of baseline biomarker differences that may influence clinical interpretation. Moreover, the simultaneous evaluation of PSA molecular forms, sex hormone balance, and hormone-to-biomarker ratios provides a broader view of ancestry- and context-related biochemical differences potentially relevant to PCa risk assessment.

## 5 Conclusion

In conclusion, our findings support the need for larger, multicentre, age-matched studies to validate whether ethnicity-conscious interpretation of PCa biomarkers may improve diagnostic accuracy and contribute to more equitable early detection. PCa disparities should be addressed not only through improved access to screening and culturally adapted prevention, but also through a more precise and equitable interpretation of the laboratory biomarkers used to guide early diagnosis.

## Authors’ contributions

Conception and design: Matteo Ferro and Daniela Terracciano. Data collection: Felice Crocetto, Francesco del Giudice, Gianluigi Carbone, Rosa Sirica, Maria Perito, Carmela Polito and Mariano Fiorenza. Data analysis and interpretation: Matteo Ferro, Daniela Terracciano. Manuscript writing: Daniela Terracciano. All authors read and approved the final version of the manuscript.

## Statement for studies involving humans and/or animals

This study was conducted in accordance with the ethical principles of the Declaration of Helsinki. The study protocol was reviewed and approved by the Ethics Committee of the University of Naples Federico II/AOU Federico II, Naples, Italy (approval code: 118/20). All participants provided written informed consent before enrolment.

No animal studies were performed by the authors.

## Funding

This study received in-kind support from Beckman Coulter in the form of reagents and diagnostic kits for PHI measurement. Beckman Coulter had no role in study design, data collection, data analysis, interpretation of results, manuscript preparation, or the decision to submit the manuscript for publication.

## Declaration of competing interest

The authors declare that they have no known competing financial interests or personal relationships that could have appeared to influence the work reported in this paper.

## Data Availability

The data supporting the findings of this study are available from the corresponding author upon reasonable request. Individual participant data are not publicly available due to privacy and ethical restrictions.
